# Integrative Single-Cell and Bulk RNA Sequencing Identifies a Macrophage-Related Prognostic Signature for Predicting Prognosis and Therapy Responses in Colorectal Cancer

**DOI:** 10.3390/ijms26020811

**Published:** 2025-01-19

**Authors:** Shaozhuo Xie, Siyu Hou, Jiajia Chen, Xin Qi

**Affiliations:** School of Chemistry and Life Science, Suzhou University of Science and Technology, Suzhou 215011, China; xsz1931206751@163.com (S.X.); 2211121003@post.usts.edu.cn (S.H.); njucjj@126.com (J.C.)

**Keywords:** colorectal cancer, single-cell RNA-seq, macrophage, prognostic signature

## Abstract

Colorectal cancer (CRC) is one of the most common malignant tumors, characterized by a high incidence and mortality rate. Macrophages, as a key immune cell type within the tumor microenvironment (TME), play a key role in tumor immune evasion and the progression of CRC. Therefore, identifying macrophage biomarkers is of great significance for predicting the prognosis of CRC patients. This study integrates scRNA-seq and bulk RNA-seq data to identify macrophage-related genes in CRC. By applying a comprehensive machine learning framework, the macrophage-related prognostic signature (MRPS) was constructed by 15 macrophage-related genes with prognostic values. The MRPS demonstrated strong predictive performance across multiple datasets, effectively stratifying high-risk and low-risk patients in terms of overall survival (OS) and disease-specific survival (DSS). Furthermore, immune analysis revealed significant differences between the high-risk and low-risk groups in immune cell infiltration levels and immune checkpoint gene expression patterns. Drug screening identified several small molecules, including Bortezomib and Mitoxantrone, as potential therapeutic options for high-risk patients. Pseudotime trajectory analysis further highlighted the potential role of genes comprising the MRPS in macrophage differentiation. This study provides a powerful tool for personalized prognosis prediction in CRC patients, offering new insights into macrophage-driven mechanisms in tumor progression and potential therapeutic strategies.

## 1. Introduction

Colorectal cancer (CRC) is one of the common malignant tumors worldwide. According to the statistical data in 2022, CRC had the third-highest incidence rate among all malignant tumors, accounting for 9.6% of cases, and was the second-leading cause of cancer-related death, responsible for 9.3% of the total mortality [1,2]. Once CRC has metastasized, the tumor spreads from the primary tumor in the colon to other organs, such as the liver, lungs, and peritoneum, significantly complicating treatment and lowering the chances of survival. As reported, the 5-year survival rate for metastatic CRC is approximately 15% [3], reflecting the aggressive nature of metastatic disease and the limitations of current therapies. Therefore, there is an urgent need to develop prognostic tools to identify high-risk patients early, tailor individualized treatment plans, and improve the survival of patients with CRC.

Tumor microenvironment (TME), the complex and dynamic environment surrounding a tumor, plays a crucial role in CRC progression, metastasis, and response to treatment [4]. It is composed of extracellular matrix components and various cell types, including cancer-associated fibroblasts (CAFs), immune cells, inflammatory cells, and mesenchymal stem cells, all of which could influence the growth and behavior of the tumor [5]. Within the TME of CRC, macrophages are a key immune cell type that can adopt distinct phenotypes in response to signals from the surrounding tumor and stromal cells. Especially, by polarizing into the pro-tumor M2 phenotype, tumor-associated macrophages (TAMs), the predominant macrophage subset in the TME, could contribute to the tumor growth, angiogenesis, metastasis, immune evasion, and chemoresistance of CRC [6,7]. These processes create a supportive environment for tumor development, highlighting macrophages as potential therapeutic targets for reprogramming or inhibition in CRC treatment [8]. For example, Popēna et al. [9] found that M2 macrophages could promote the growth and invasion of CRC by releasing chemokines such as IL-17 and EGFR. Similarly, Mola et al. [10] observed that high infiltration of M1 macrophages at the tumor invasive front is significantly associated with improved prognosis in CRC patients. Therefore, macrophages play a crucial role in CRC progression and are key determinants of clinical outcomes.

**Figure 1:**
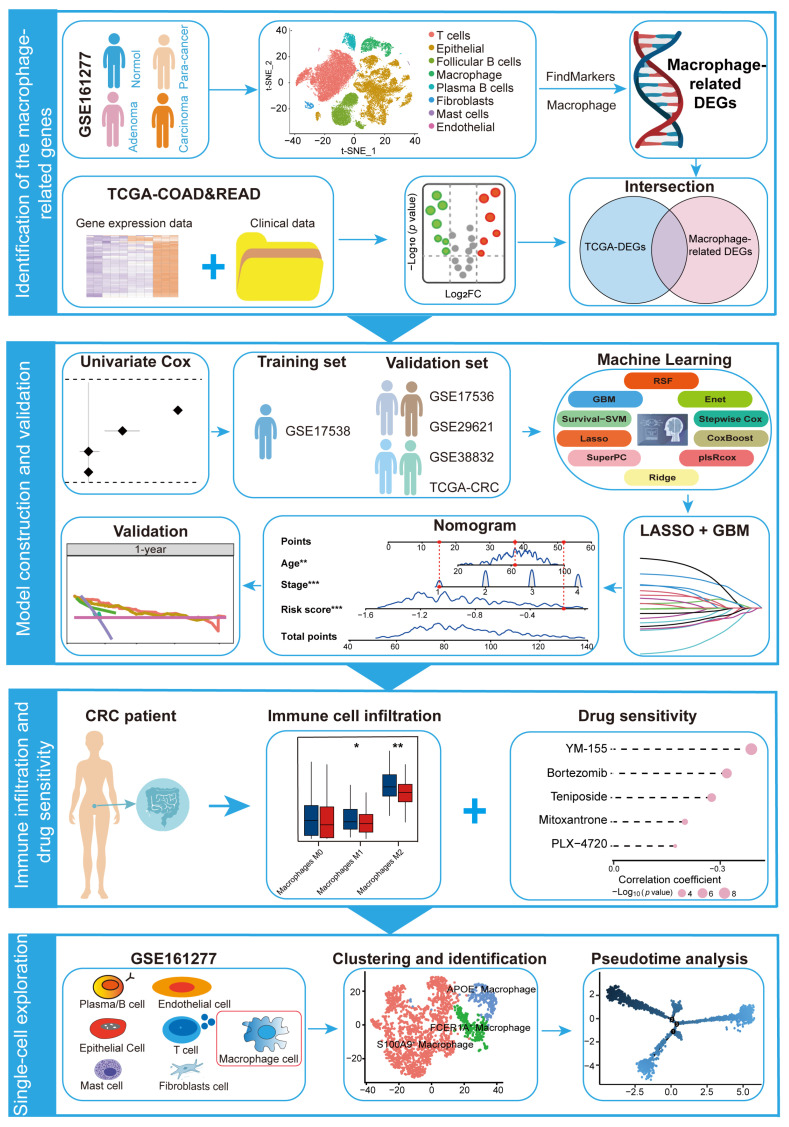
The schematic diagram of the analysis workflow for this study. * *p* < 0.05; ** *p* < 0.01; *** *p* < 0.001.

Given the pivotal role of macrophages in shaping the TME and modulating immune responses, identifying macrophage-specific biomarkers is crucial for predicting the survival of CRC patients. Currently, advancements in single-cell RNA-seq (scRNA-seq) technology have revolutionized cancer research, allowing us to analyze gene expression patterns, and discover distinct cell populations and their associated biomarkers at the single-cell level [11]. This study integrated scRNA-seq and bulk RNA-seq data to systematically identify macrophage-related prognostic biomarkers. The MRPS was developed by using a comprehensive machine learning framework that combines 101 distinct algorithm combinations. The predictive performance of the MRPS was evaluated through Kaplan–Meier (KM) survival curve and receiver operating characteristic (ROC) curve analyses in both the training and multiple validation datasets. Additionally, a nomogram incorporating the MRPS was constructed to provide a quantitative tool for clinical prognosis prediction. The study also assessed the response of different risk groups to immune therapy and identified drugs targeting specific subgroups. The results demonstrated that the MRPS effectively predicted the prognosis of CRC patients and was closely associated with their response to drug treatments, laying a foundation for further mechanistic studies and clinical applications. The analysis workflow for this study is illustrated in Figure 1.

## 2. Results

### 2.1. Identification of Macrophage-Related Genes in CRC Through scRNA-Seq Analysis

**Figure 2:**
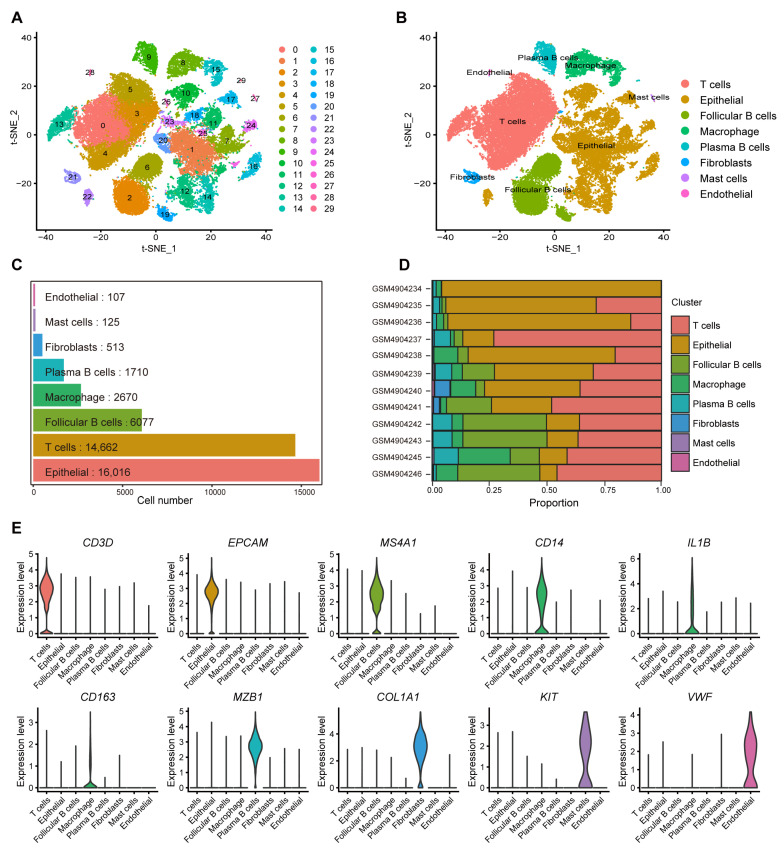
scRNA-seq analysis of CRC tissues. (**A**,**B**) The t-SNE diagram shows 29 cell clusters (**A**) and their corresponding cell types (**B**). (**C**,**D**) The number (**C**) and composition ratio (**D**) of eight different cell types in 12 samples. (**E**) Expression levels of recognized marker genes in eight cell types.

To explore macrophage-related genes in CRC, scRNA-seq dataset GSE161277, comprising a total of 48,381 cells from 12 samples, was analyzed. After stringent quality control, 41,880 high-quality cells were retained for further analysis (Figure S1A,B). Firstly, PCA was performed on the top 2000 most variable genes, and batch effects were corrected using the Harmony method. Dimensionality reduction was then conducted using the t-distributed stochastic neighbor embedding (t-SNE) method, and cells were clustered at a resolution of 0.9, resulting in 29 distinct clusters (Figure 2A). Based on the expression patterns of canonical marker genes, these clusters were further annotated into eight major cell types (Figure 2B,E): T cells (*CD3D*), epithelial cells (*EPCAM*), follicular B cells (*MS4A1*), plasma B cells (*MZB1*), macrophages (*CD14*, *IL1B*, *CD163*), fibroblasts (*COL1A1*), mast cells (*KIT*), and endothelial cells (*VWF*). Notably, the scRNA-seq dataset GSE161277 included 2670 macrophages (Figure 2C). Figure 2D further illustrates the distribution of macrophages across the 12 samples, along with the proportions of T cells, epithelial cells, follicular B cells, macrophages, plasma B cells, fibroblasts, mast cells, and endothelial cells in each sample, highlighting the cellular heterogeneity within the TME. These samples were derived from different pathological conditions, including normal tissues (GSM4904237, GSM4904240, and GSM4904246), adenoma tissues (GSM4904235, GSM4904238, GSM4904242, and GSM4904243), carcinoma tissues (GSM4904234, GSM4904236, GSM4904239, and GSM4904245), and para-cancer tissues (GSM4904241). The results indicate that macrophages are widely present across all sample types, from normal tissues to tumor tissues. Additionally, 1193 genes specifically expressed in macrophages were identified using the FindMarkers function. Therefore, this analysis identified eight major cell types and their corresponding key biomarkers.

### 2.2. Identification of Macrophage-Related Genes with Prognostic Significance

**Figure 3:**
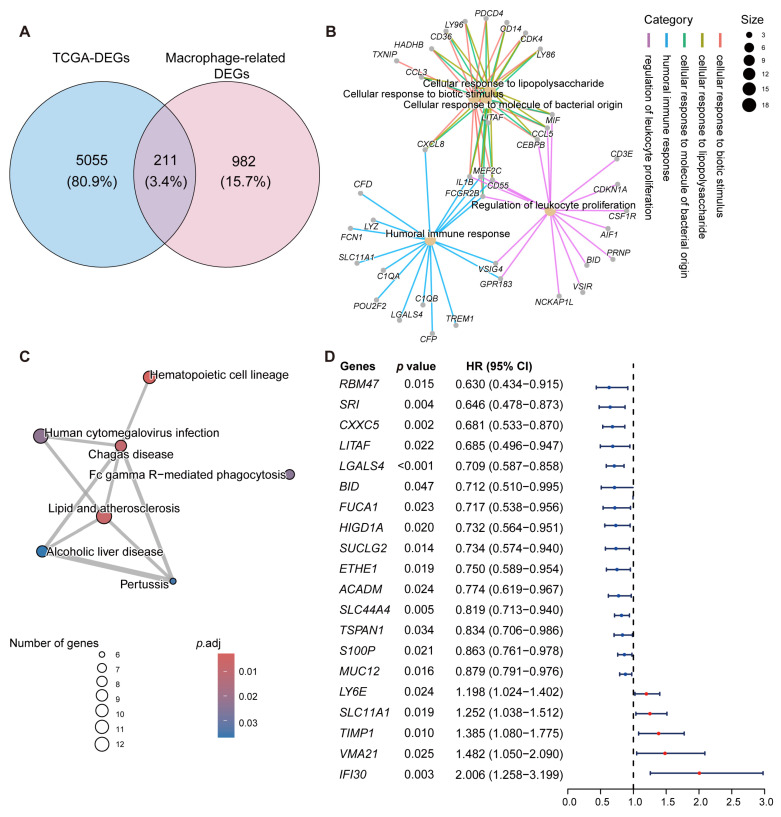
Identification of macrophage-related genes with prognostic significance. (**A**) Venn plot showing the overlapping genes between DEGs identified in TCGA-CRC dataset and macrophage marker genes identified in the GSE161277 dataset. (**B**,**C**) GO (**B**) and KEGG (**C**) enrichment analysis of macrophage-related genes identified in CRC. (**D**) Macrophage-related genes with prognostic value were identified by univariate Cox regression analysis.

To identify key genes involved in CRC development, differential expression analysis was performed on normal and tumor samples from the TCGA-CRC dataset using the “DESeq2” R package (version 1.42.1). A total of 5266 DEGs were identified based on the criteria of |log_2_(FC)| > 1 and *p*.adj < 0.05. Subsequently, by taking the intersection of macrophage-specific genes from the scRNA-seq dataset with DEGs from the bulk RNA-seq dataset, 211 macrophage-related genes were identified in CRC (Figure 3A). As shown in Figure 3B, those macrophage-related genes in CRC were mainly enriched in immune-related biological process items, including “Cellular response to biotic stimulus”, “Cellular response to lipopolysaccharide”, “Cellular response to molecule of bacterial origin”, “Humoral immune response”, and “Regulation of leukocyte proliferation”. In addition, those macrophage-related genes in CRC were significantly involved in KEGG pathways, such as “Hematopoietic cell lineage”, “Lipid and atherosclerosis”, “Chagas disease”, “Human cytomegalovirus infection”, “Fc gamma R-mediated phagocytosis”, “Pertussis”, and “Alcoholic liver disease” (Figure 3C). Moreover, 20 macrophage-related genes with prognostic significance in CRC were identified through univariate Cox regression analysis (Figure 3D).

### 2.3. Construction of 15-Gene MRPS Based on Machine Learning

**Figure 4:**
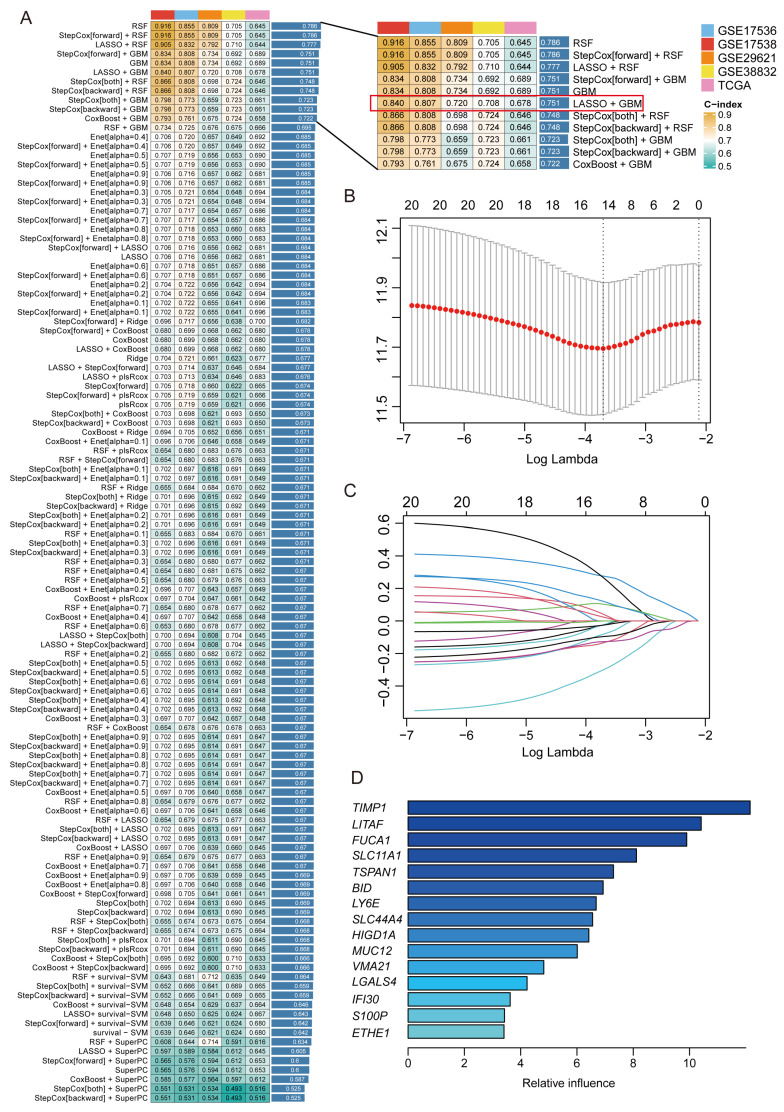
Construction of 15-gene MRPS based on machine learning approaches. (**A**) A total of 101 algorithm combinations were employed to construct prognostic models in CRC and the C-index value of each model was subsequently calculated across all validation datasets. (**B**) Calculation of the optimal lambda value. (**C**) Determination of the coefficients of independent variables by LASSO Cox regression analysis. (**D**) The regression coefficients for the 15 genes identified in the GBM.

Based on the 20 macrophage-related genes with prognostic significance in CRC identified from the scRNA-Seq dataset GSE161277, 101 machine learning algorithms were applied to the training dataset GSE17538, and multiple validation datasets including GSE17536, GSE29621, GSE38832, and TCGA-CRC were used to assess the generalizability of the prognostic models. As shown in Figure 4A, the prognostic models constructed by RSF, StepCox (forward) + RSF, and LASSO + RSF achieved the top three highest average concordance index (C-index) values across all the cohorts. However, due to the limitation of the RSF algorithm, these RSF-based models exhibited a high error rate of 40% and performed poorly in the validation datasets. Comparatively, the models developed by StepCox (forward) + GBM, GBM, and LASSO + GBM achieved the identical average C-index value, ranking just below the top three models. Notably, the models constructed by StepCox (forward) + GBM or GBM incorporated all 20 macrophage-related genes, while the LASSO + GBM model comprised only 15 genes yet achieved comparable predictive performance (Figure 4B–D). Accordingly, the LASSO + GBM algorithm combination was chosen to construct the MRPS due to its ability to select fewer genes during feature selection. In detail, in the LASSO Cox regression analysis, 15 genes with significant non-zero coefficients were identified using the optimal λ value of 0.02468. These genes were considered to have an important impact on prognosis and were subsequently used in the GBM analysis (Figure 4B–D). For each patient, a risk score was calculated based on the expression level of each gene and its corresponding coefficient: risk score = (0.119802 × expression of *HIGD1A*) + (−0.299944 × expression of *LITAF*) + (−0.127631 × expression of *SLC44A4*) + (−0.025520 × expression of *MUC12*) + (0.023400 × expression of *IFI30*) + (−0.023745 × expression of *BID*) + (−0.133584 × expression of *SLC11A1*) + (0.275531 × expression of *TIMP1*) + (−0.023084 × expression of *ETHE1*) + (0.264840 × expression of *TSPAN1*) + (0.040592 × expression of *VMA21*) + (0.102673 × expression of *LY6E*) + (−0.080988 × expression of *S100P*) + (−0.096006 × expression of *LGALS4*) + (−0.000066 × expression of *FUCA1*) (Table S1). The genes comprising the MRPS were referred to as MRPS-related genes.

### 2.4. Performance Evaluation and Validation of MRPS in CRC

**Figure 5:**
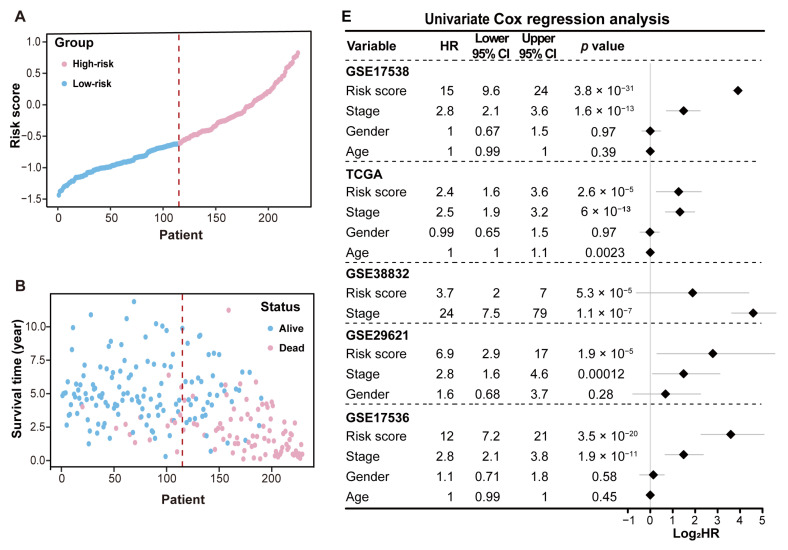

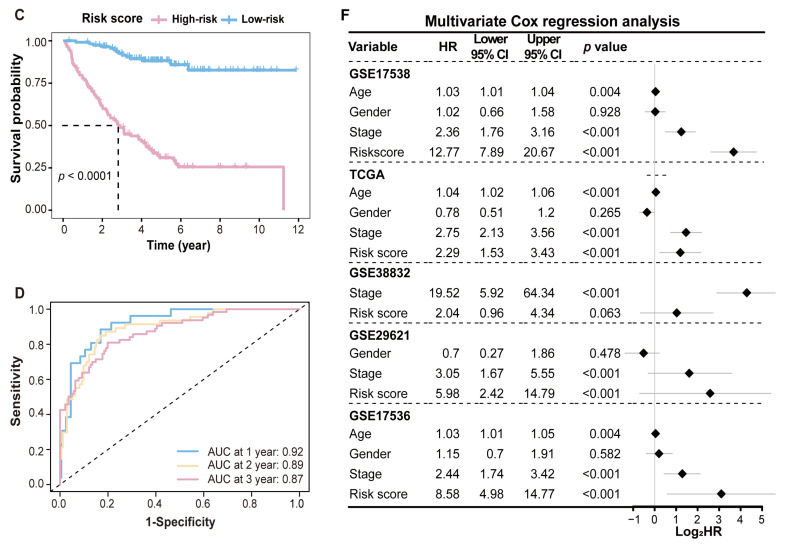
Performance evaluation and validation of the MRPS in predicting the survival of CRC patients. (**A**) Distribution of the patients in low- and high-risk groups in the GSE17538 dataset. The dashed line indicates the cutoff for categorizing CRC patients into high-risk and low-risk groups based on the median risk score. (**B**) Survival status for each patient in the GSE17538 dataset. The dashed line indicates the cutoff for categorizing CRC patients into high-risk and low-risk groups based on the median risk score. (**C**) KM survival curves for OS of CRC patients in the high-risk and low-risk groups in the GSE17538 dataset. The dashed lines indicate the survival time (vertical lines) at which the survival probability reaches 0.50 (horizontal lines) for patients in the high-risk group. (**D**) ROC curves show the specificity and sensitivity of the MRPS in predicting OS at 1, 2, and 3 years in the GSE17538 dataset. The dashed line in the diagonal represents the ROC curve of a random predictor, with an AUC of 0.5. (**E**) Univariate Cox regression analysis of the clinical features and the risk score in patients with CRC. (**F**) Multivariate Cox regression analysis of the clinical features and the risk score in patients with CRC.

To evaluate the MRPS model’s predictive potential for overall survival (OS) and disease-specific survival (DSS), CRC patients in each cohort were stratified into high-risk and low-risk groups based on the median risk score (Figure 5A). Patients in the high-risk group had shorter survival times and a higher proportion of deaths compared to those in the low-risk group (Figure 5B). The KM analysis across the training and validation sets demonstrated that the low-risk group had significantly longer OS or DSS (log-rank test, *p* < 0.01) (Figure 5C and Figure S2A–D). The ROC curve analysis revealed the area under curve (AUC) values for predicting 1-, 2-, and 3-year OS in the GSE17538 training dataset to be 0.92, 0.89, and 0.87, respectively (Figure 5D). In the validation cohorts, the AUC values for the corresponding intervals were 0.90, 0.86, and 0.82 in the GSE17536 dataset; 0.87, 0.77, and 0.69 in the GSE29621 dataset; 0.76, 0.71, and 0.71 in the GSE38832 dataset; and 0.70, 0.67, and 0.66 in the TCGA-CRC dataset (Figure S2E–H). To further evaluate the accuracy and reliability of the MRPS, its performance was compared with 74 previously published prognostic models in CRC (Table S2). Notably, the MRPS exhibited the highest C-index values across the GSE17538, GSE17536, GSE29621, and GSE38832 datasets, and outperformed most of the published models in the TCGA-CRC dataset (Figure S2I–M). These findings suggest that the MRPS has strong discriminative ability, highlighting its utility in predicting survival outcomes for CRC patients.

In addition, to assess the independent capability of the MRPS in predicting OS or DSS, univariate and multivariate Cox regression analyses were conducted across five datasets: GSE17538, GSE17536, GSE29621, GSE38832, and TCGA-CRC. Univariate analysis showed that the risk score and clinical stage had Hazard ratios (HRs) greater than 1 across all datasets (*p* < 0.05), indicating a significant impact on CRC survival (Figure 5A). Multivariate Cox regression analysis confirmed the risk score as an independent prognostic factor in the GSE17538, GSE17536, GSE29621, and TCGA-CRC cohorts (*p* < 0.05). However, in the GSE38832 dataset, although the HR for the risk score was greater than 1, the *p*-value was not significant (*p* > 0.05), indicating that the model cannot be used as an independent prognostic factor for DSS in CRC (Figure 5B).

### 2.5. Establishment and Validation of an MRPS-Based Nomogram for CRC

**Figure 6:**
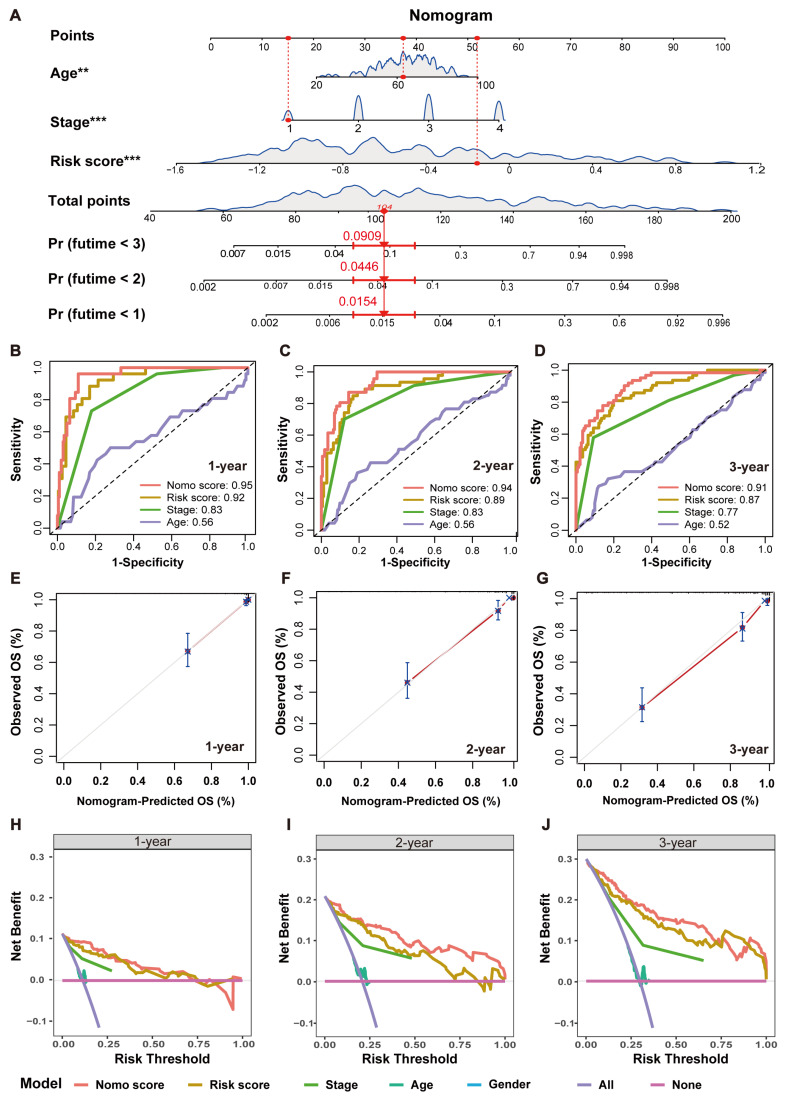
Establishment and validation of an MRPS-based nomogram for CRC in the GSE17538 dataset. (**A**) The nomogram was constructed by combining clinical features with risk score. (**B**–**D**) Multivariate ROC curves of nomograms for predicting 1-year (**B**), 2-year (**C**), and 3-year (**D**) OS of CRC patients. (**E**–**G**) Calibration curves of nomograms for predicting 1-year (**E**), 2-year (**F**), and 3-year (**G**) survival probabilities of CRC patients in the GSE17538 dataset. (**H**–**J**) Clinical decision curves of nomograms for predicting 1-year (**H**), 2-year (**I**), and 3-year (**J**) survival probabilities of CRC patients in the GSE17538 dataset. ** *p* < 0.01; *** *p* < 0.001.

To enhance the clinical applicability of the MRPS, we constructed a nomogram incorporating the MRPS-derived risk score and clinical characteristics such as age and stage (Figure 6A). As shown in Figure 6B–D, the nomogram achieved AUC values of 0.95, 0.94, and 0.91 at 1-year, 2-year, and 3-year intervals, respectively, indicating its high predictive accuracy. The calibration curves show good agreement between the nomogram’s predictions and actual observations (Figure 6E–G). In addition, decision curve analysis (DCA)indicated that the nomogram could provide better net clinical benefit than other clinical characteristics (Figure 6H–J). These findings highlight the potential of a nomogram as a reliable and accurate tool for personalized prognosis prediction in CRC patients.

### 2.6. Immune Landscape Variations Between High- and Low-Risk Groups in CRC

**Figure 7:**
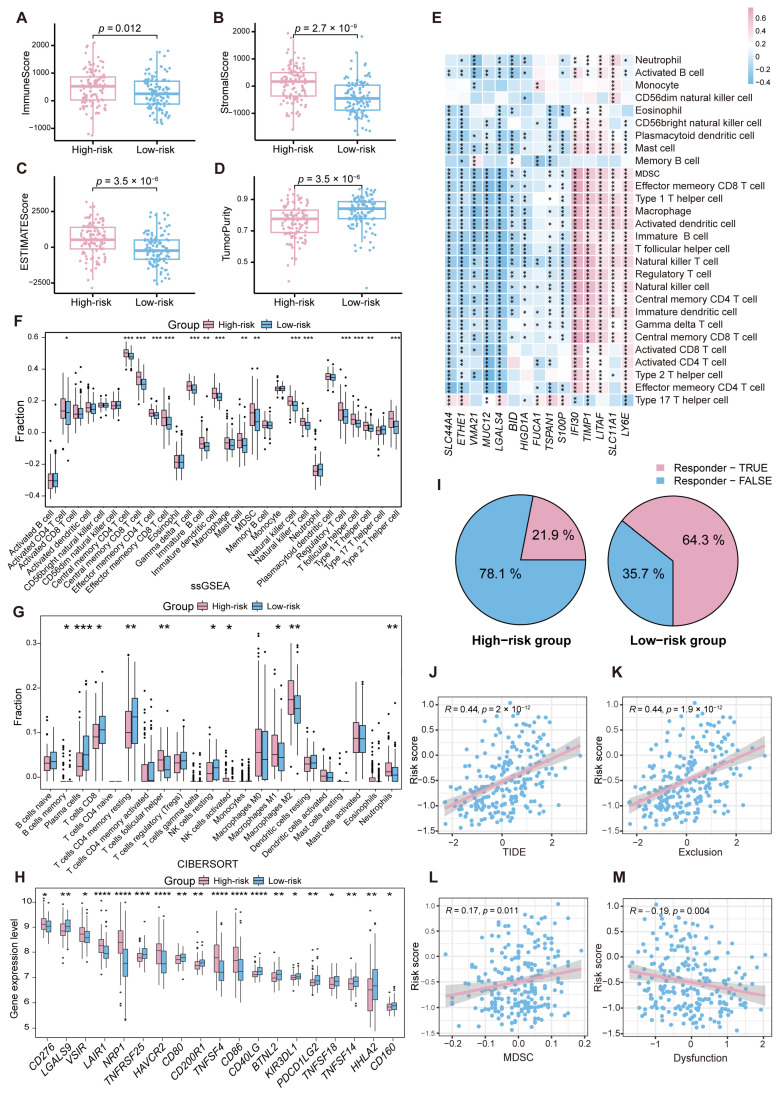
Immune landscape variations between high- and low-risk groups in CRC. (**A**–**D**) The immune score (**A**), stromal score (**B**), tumor purity (**C**), and ESTIMATE score (**D**) were applied to quantify the different immune status between the high-risk and low-risk groups. (**E**) Correlation between the expression levels of the 15 MRPS-related genes and the levels of immune cell infiltration. (**F**,**G**) The abundance of each TME-infiltrated cell type between the high- and low-risk groups, quantified by the ssGSEA algorithm (**F**) and CIBESORT algorithm (**G**). (**H**) The expression levels of 19 immune checkpoint genes between the high-risk and low-risk groups. (**I**) Proportion comparison of CRC patients responding to immunotherapy between the high-risk and low-risk groups. (**J**–**M**) Association of risk score with TIDE score (**J**), exclusion score (**K**), MDSC cell score (**L**), and dysfunction score (**M**). * *p* < 0.05; ** *p* < 0.01; *** *p* < 0.001; **** *p* < 0.0001.

To explore the immune landscape variations between high-risk and low-risk groups in CRC, the ESTIMATE algorithm was employed to calculate the stromal score, immune score, estimate score, and tumor purity score for each sample in the GSE17538 dataset. As shown in Figure 7A–C, significant differences were observed in immune scores, stromal scores, and ESTIMATE scores between the high-risk and low-risk groups, with the high-risk group exhibiting higher scores (*p* < 0.05). In addition, the tumor purity score in the high-risk group was relatively lower, suggesting a more complex TME in this group (Figure 7D). Next, the single-sample gene set enrichment analysis (ssGSEA) algorithm was used to quantify immune cell infiltration levels. The results showed that MRPS-related genes were significantly correlated with key immune active cell types, including macrophages, effector memory CD4^+^ T cells, and neutrophils (Figure 7E). This highlights the potential role of MRPS-related genes in shaping immune infiltration patterns and regulating the TME. Furthermore, in the high-risk group, the infiltration levels of several immune cell types, including effector memory CD4^+^ T cells, central memory CD4^+^ T cells, activated B cells, and neutrophils, were significantly higher compared to the low-risk group (Figure 7F). Additional analysis using the CIBERSORT algorithm revealed that M1 and M2 macrophages were more abundant in the high-risk group, with significant differences observed when compared to the low-risk group (Figure 7G). Notably, the expression levels of *IFI30*, *TIMP1*, *LITAF*, *SLC11A1*, and *LY6E* that constitute the MRPS exhibited a positive correlation with the infiltration level of macrophages (Figure 7E).

Furthermore, the immunotherapy response was compared between the high-risk and low-risk groups of CRC patients. As shown in Figure 7H, there were significant differences in the mRNA expression levels of 19 immune checkpoint genes between the high-risk and low-risk groups. Subsequently, the differences in immunotherapy responses between the high-risk and low-risk groups were evaluated. It shows that the proportion of responders was higher in the low-risk group compared to the high-risk group (Figure 7I). In addition, significant differences were observed between the two groups in TIDE scores, dysfunction scores, exclusion scores, and MDSC cell scores, with the TIDE, exclusion, and MDSC cell scores positively correlated with the risk score (Figure 7J–L), whereas the dysfunction scores were negatively correlated with the risk score (Figure 7M). These findings suggest that high-risk patients are more likely to suffer immune evasion and may benefit less from immunotherapy.

### 2.7. Impact of TMB on the Survival of CRC Patients with Distinct Risks

**Figure 8:**
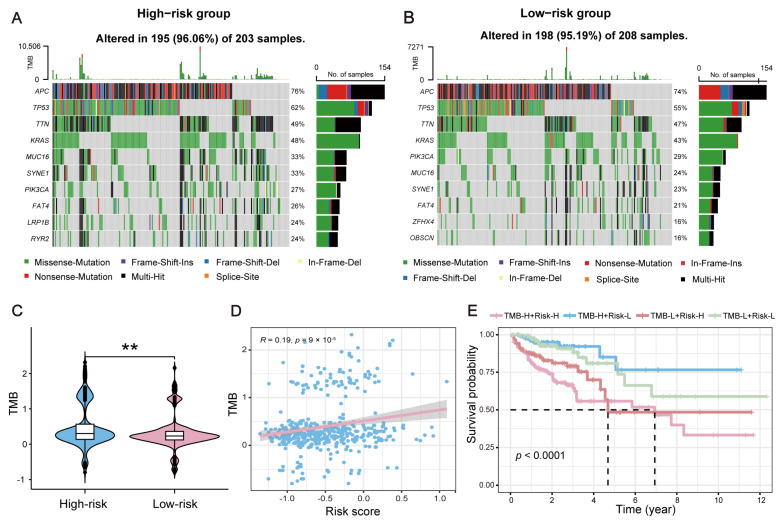
Impact of TMB on the survival of CRC patients with distinct risks. (**A**) Top 10 genes with the highest mutation rates in the high-risk group. (**B**) Top 10 genes with the highest mutation rates in the low-risk group. (**C**) Analysis of the difference in TMB values between the high-risk and low-risk groups. (**D**) Correlation analysis between TMB values and risk scores. (**E**) The KM curves for four CRC patient groups: TMB-H + Risk-H, TMB-H + Risk-L, TMB-L + Risk-H, and TMB-L + Risk-L. The dashed lines indicate the survival time (vertical lines) at which the survival probability reaches 0.50 (horizontal lines) for patients in the TMB-H + Risk-H and TMB-L + Risk-H groups. ** *p* < 0.01.

To investigate the mutation status across different risk groups, we analyzed the differences in the tumor mutation burden (TMB) between the high-risk and low-risk groups in the TCGA-CRC dataset and performed a combined survival analysis incorporating the TMB values and risk group stratification. As shown in Figure 8A,B, the overall levels of TMB were significantly higher in the high-risk group compared to the low-risk group. In addition, the key genes, such as TP53 and KRAS, had a significantly higher mutation frequency in the high-risk group. Consistently, high-risk patients exhibited significantly higher TMB values than low-risk patients (*p* < 0.01, Figure 8C,D). Moreover, KM survival analysis was performed by dividing CRC patients into four subgroups: TMB-H + Risk-H, TMB-H + Risk-L, TMB-L + Risk-H, and TMB-L + Risk-L. As shown in Figure 8E, patients in the TMB-H + Risk-H subgroup had significantly worse prognosis compared to those in the TMB-L + Risk-L subgroups (*p* < 0.001).

### 2.8. Screening of Potential Drugs for CRC Patients

**Figure 9:**
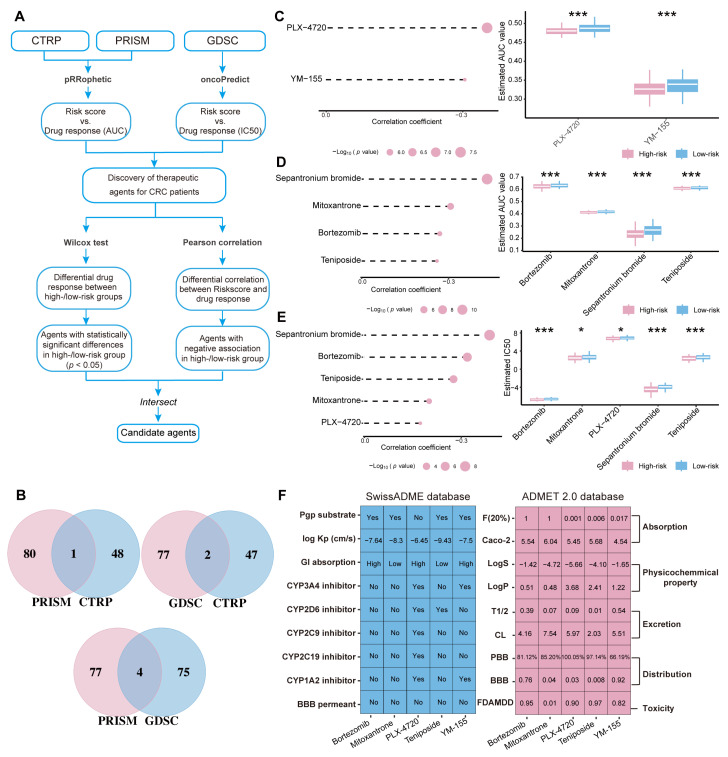
Identification of potential therapeutic drugs for patients with CRC. (**A**) Flowchart of the process for screening potential therapeutic drugs for CRC based on the GDSC, CTRP, and PRISM databases. (**B**) Intersection of potential therapeutic compounds screened from the GDSC, CTRP, and PRISM databases. (**C**) Screening outcomes for potential therapeutic drugs (PLX-4720 and YM-155) in the CTRP database. (**D**) Screening outcomes for potential therapeutic drugs (YM-155, Mitoxantrone, Bortezomib, Teniposide) in the PRISM database. (**E**) Screening outcomes for potential therapeutic drugs (YM-155, Bortezomib, Teniposide, Mitoxantrone, PLX-4720) in the GDSC database. (**F**) Analysis of the ADMET properties of potential therapeutic drugs using the ADMETlab 2.0 and SwissADME databases. * *p* < 0.05; *** *p* < 0.001.

To predict potential drugs with significantly different sensitivities between high-risk and low-risk patient groups, two approaches were utilized to integrate gene expression data with drug sensitivity data. Firstly, the Wilcoxon rank-sum test was used to compare drug sensitivity differences between the two groups, using a statistical significance threshold of *p* < 0.05. Next, Pearson correlation analysis was performed to evaluate the relationship between drug response (measured by IC50 or AUC values) and risk scores, identifying drugs significantly correlated with the risk scores. Based on these selection criteria, 79, 49, and 81 drugs were identified from the Genomics of Drug Sensitivity in Cancer (GDSC), Cancer Therapeutics Response Portal (CTRP), and PRISM databases, respectively (Figure 9A). Subsequently, a Venn diagram was used to determine the overlap of drug candidates across these databases (Figure 9B). Notably, five drugs identified in at least two databases, including Sepantronium bromide (YM-155), Bortezomib, Teniposide, Mitoxantrone, and PLX-4720, were considered promising candidates for CRC treatment (Figure 9C–E).

The absorption, distribution, metabolism, excretion, and toxicity (ADMET) properties of these drugs were evaluated using the SwissADME and ADMETlab 2.0 databases. According to the analysis from SwissADME, among the five drugs, Bortezomib exhibits a relatively high gastrointestinal (GI) absorption rate, and it is not an inhibitor of several cytochrome P450 (CYP) enzymes. The results from the ADMET 2.0 database further support Bortezomib’s favorable ADMET characteristics, including an F (20%) score of 1 and relatively high solubility. Similarly, Mitoxantrone does not inhibit any CYP enzymes, with an F (20%) score of 1 and a high clearance rate. In contrast, PLX-4720 and Teniposide have poorer absorption, and YM-155 inhibits CYP enzymes, which may limit their therapeutic efficacy (Figure 9F). Overall, both Bortezomib and Mitoxantrone exhibit relatively good absorption and metabolic characteristics.

### 2.9. Exploring the Role of MRPS-Related Genes in Macrophage Development via Pseudotime Trajectory Analysis

**Figure 10:**
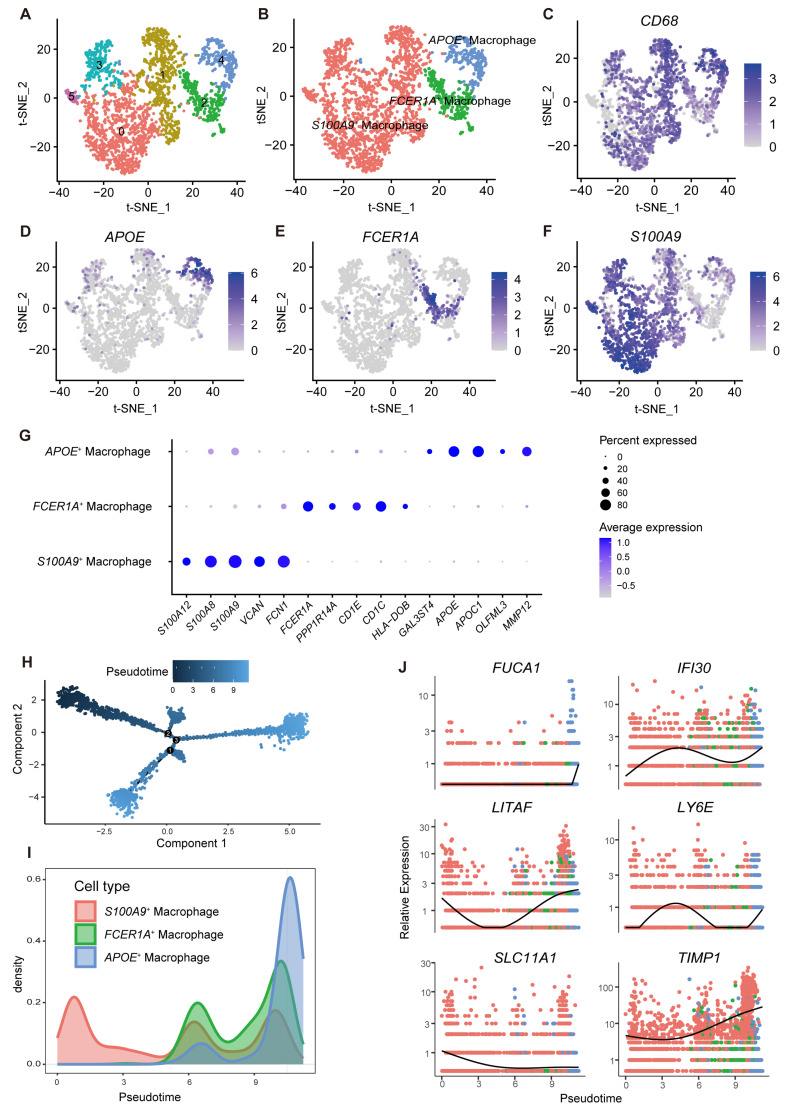
Pseudotime trajectory analysis of macrophage subclusters. (**A**) The t-SNE plot of macrophages in CRC. Cells were clustered into six clusters and colored by clusters. (**B**) The t-SNE plot of macrophage subtypes in CRC. (**C**–**F**) The t-SNE plots showed expression levels of *CD68*, *APOE*, *FCER1A*, and *S100A9* in macrophages. (**G**) Dot plot showed top five marker genes of *APOE*^+^, *FCER1A*^+^, and *S100A9*^+^ macrophages. (**H**) Pseudotime plot showed the developmental trajectory of macrophages, with colors ranging from dark blue to light blue indicating pseudotime scores from low to high. (**I**) The density plot showed pseudotime trajectory of the *APOE*^+^, *FCER1A*^+^, and *S100A9*^+^ macrophage subclusters. (**J**) The expression levels of *FUCA1*, *IFI30*, *LITAF*, *LY6E*, *SLC11A1*, and *TIMP1* along the pseudotime trajectory across the *APOE*^+^, *FCER1A*^+^, and *S100A9*^+^ macrophage subclusters. The colors correspond to the three macrophage subpopulations: red for *S100A9*^+^ macrophages, green for *FCER1A*^+^ macrophages, and blue for *APOE*^+^ macrophages.

To investigate the potential role of MRPS-related genes in the development of macrophages, clustering analysis was performed on the macrophages identified in the GSE161277 dataset. These macrophages were grouped into six distinct clusters (Figure 10A). Based on the expression patterns of marker genes, these six clusters were further annotated as three cell types: *APOE*^+^ macrophages, *FCER1A*^+^ macrophages, and *S100A9*^+^ macrophages (Figure 10B). The FeaturePlot function was used to visualize the expression of known macrophage-specific marker genes (Figure 10C–F). Notably, the feature plot of *CD68* gene expression confirmed that all cells within the clustered groups were macrophages. The expression levels of the top five genes in each of the three subgroups were illustrated in a dot plot, further validating the accuracy of the macrophage subpopulation identification (Figure 10G).

To further explore the impact of MRPS-related genes on macrophage development, single-cell pseudotime trajectory analysis was performed on these subpopulations. The results revealed that the *S100A9*^+^ macrophage was located at the starting position of the trajectory path, while the *FCER1A*^+^ and *APOE*^+^ macrophages were at the terminal states of the trajectory (Figure 10H,I and Figure S3A,B). Analysis of the expression levels of 15 MRPS-related genes along the pseudotime trajectory revealed that six genes (*SLC11A1*, *FUCA1*, *TIMP1*, *LITAF*, *IFI30*, *LY6E*) exhibited dynamic changes, while the remaining nine genes displayed relatively stable expression with no significant variation throughout the trajectory (Figure 10J and Figure S3C). These findings revealed that the dynamic changes in the expression of these genes are closely linked to the differentiation process of macrophages, transitioning from the early proliferative phase to the later stage of functional specialization. This highlights the significant role of MRPS-related genes in macrophage differentiation and functional transition.

## 3. Discussion

Recently, the progress of scRNA-seq allows for the analysis of gene expression at the individual cell level, revealing subtle variations that could be critical for understanding cancer progression and patient prognosis. Combining bulk and single-cell transcriptome data could not only improve the accuracy of prognostic models by capturing both the broad tumor landscape and the detailed cellular interactions within the TME but also open the door to more personalized treatment strategies and novel therapeutic targets. In this study, by integrating scRNA-seq and bulk RNA-seq data, we successfully constructed and validated an MRPS consisting of 15 genes, using the combination of LASSO and GBM algorithms. To address the potential risk of overfitting, we applied ten-fold cross-validation during training, validated the model on multiple independent datasets, and used LASSO for feature selection to reduce model complexity. The consistent performance across validation datasets suggests the model’s robustness. Notably, the MRPS serves as an independent prognostic factor and can accurately predict the OS of CRC patients. To enhance the predictive performance of this signature and improve its clinical applicability, a nomogram was subsequently developed based on age, stage, and risk score, offering a clinically practical tool for predicting OS (Figure 6). These findings highlight the potential of integrating single-cell and bulk RNA-seq data to improve prognostic modeling and provide valuable tools for developing personalized treatment strategies in CRC.

The analysis of immune landscape variations between high-risk and low-risk CRC groups revealed distinct TME characteristics influencing prognosis and therapy responses. The high-risk group exhibited higher stromal and immune scores, lower tumor purity, and increased infiltration of immunosuppressive cells, including M2 macrophages and MDSCs, along with upregulated immune checkpoint gene expression, indicating a more immunosuppressive environment prone to immune evasion. Studies have shown that M2 macrophages suppress the functions of immune cells such as Th1 and Th2 CD4^+^ T cells, cytotoxic T lymphocytes, and NK cells by secreting anti-inflammatory factors like IL-10 and TGF-β [12,13]. This suppression could weaken the immune system’s ability to recognize and attack tumor cells, creating an immune-escape microenvironment that promotes tumor cell proliferation and invasion [14]. Additionally, M2 macrophages can secrete angiogenic factors such as VEGF, which facilitate angiogenesis [15], thereby supporting distant metastasis of tumor cells and accelerating the progression of CRC [16]. Similarly, MDSCs could not only inhibit the function of effector immune cells but also promote tumor angiogenesis and metastasis [17,18]. Joshi et al. [18] demonstrated that a high infiltration level of MDSCs was associated with poor prognosis in CRC and various other cancers. In contrast, the low-risk group demonstrated higher infiltration of immune-active cells, such as effector memory CD4^+^ T cells and activated B cells. Bos et al. [19] reported that CD4^+^ T cells significantly enhance the recruitment of CD8^+^ T cells by secreting IFN-γ-induced chemokines [20]. Furthermore, CD4^+^ T cells could inhibit tumor cell cycle progression and activate pro-inflammatory signaling pathways involving TNF and INF-γ, effectively inducing tumor cell regression [21]. Activated B cells also play a critical role by inducing Th1-type CD4^+^ T cell responses [22], working synergistically to bolster tumor immune responses. TIDE analysis further confirmed that high-risk patients are more likely to experience immune escape and reduced immunotherapy efficacy. These findings underscore the need for tailored therapeutic strategies targeting the immunosuppressive microenvironment in high-risk CRC patients to enhance treatment outcomes. Overall, the distinct immune landscapes between high- and low-risk groups highlight the critical influence of the TME on CRC progression and provide insights into potential immunotherapeutic strategies tailored to different patient subgroups.

To better guide the treatment of CRC, potential small-molecule drugs for high-risk and low-risk groups of CRC patients were selected based on drug sensitivity data from databases. Five drugs selected from the GDSC, CTRP, and PRISM databases, including (YM-155, Bortezomib, Teniposide, Mitoxantrone, and PLX-4720) exhibited more significant therapeutic effects in the high-risk group (Figure 9C). As reported, Qian et al. [23] found that Mitoxantrone could reprogram the TME through inducing immunogenic cell death, thereby enhancing the anti-tumor immune response. Hong et al. [24] found that Bortezomib could induce the generation of intracellular reactive oxygen species (ROS) and ATM phosphorylation, leading to G2-M phase arrest in CRC cells, highlighting its potential in cell cycle blockage and anti-cancer therapy. Liao et al. [25] found that Teniposide can promote anti-cancer immune responses and induce cancer cell apoptosis through activation of the cGAS-STING pathway, indicating its role in enhancing anti-cancer immunity. Zhan et al. [26] demonstrated that YM-155 induces endoplasmic reticulum stress and triggers apoptosis in CMS1 subtype CRC cells. PLX-4720 inhibits cell proliferation and induces apoptosis in CRC cells harboring the BRAFV600E mutation [27]. ADMET property analysis further revealed that Bortezomib and Mitoxantrone demonstrate favorable absorption and metabolic properties. Therefore, Bortezomib and Mitoxantrone could serve as promising therapeutic options for high-risk CRC patients.

In addition, scRNA-seq analysis further classified macrophages into three subpopulations: APOE^+^, FCER1A^+^, and S100A9^+^. Single-cell trajectory analysis clearly depicted their differentiation paths, starting from S100A9^+^ macrophages and ultimately differentiating into APOE^+^ macrophages [28,29]. During this process, dynamic expression changes in MRPS-related genes were closely associated with the differentiation of macrophages from proliferation to functional specialization. For example, the expression level of the SLC11A1 gene shows a downward trend. Wyllie et al. [30] found that SLC11A1 primarily regulates iron metabolism in macrophages and plays a key role in macrophage activation during the early stages. Comparatively, the expression levels of FUCA1 and TIMP1 increased in the later stages of macrophage differentiation. Xu et al. [31] discovered that the loss of FUCA1 in gliomas significantly reduces the recruitment of TAMs to the tumor site. In CRC, the upregulation of FUCA1 expression during macrophage development may promote the polarization of macrophages toward an immunosuppressive phenotype (e.g., M2 type) [32]. TIMP1 has been shown to promote macrophage migration and inhibit the expression of M1 macrophage markers (e.g., ARG1 and CD163) [33]. The increased expression of TIMP1 may facilitate the conversion of macrophages to an immunosuppressive phenotype, thereby promoting tumor cell invasion and immune evasion. Therefore, these findings highlight the potential role of MRPS-related genes in the differentiation and functional transition of macrophages, which in turn influence the TME and patient prognosis.

However, there are still some limitations in this study. Firstly, the scRNA-seq data were obtained from only 12 CRC patients, which constitutes a small sample size, and there was significant variation in the proportions of cell subpopulations between patients. This may affect the comprehensive characterization of CRC cells. Secondly, although we evaluated and validated the MRPS in both the training and validation datasets, a large-scale, multi-center prospective study is needed to further confirm our findings. Thirdly, this study primarily relied on gene expression correlation analysis and lacked functional validation of key genes and cell types involved in tumor initiation, progression, and treatment response. Future research could expand the sample size and conduct more extensive functional studies to further validate the application potential of MRPS in CRC and other cancer types. Addressing these limitations in future studies will be crucial for fully validating the clinical utility of the MRPS and expanding its applicability in CRC and beyond.

## 4. Materials and Methods

### 4.1. Data Collection and Processing

The scRNA-seq dataset GSE161277 (*n* = 13) from four untreated CRC patients with microsatellite stability was downloaded from the Gene Expression Omnibus (GEO) dataset [34]. Twelve samples were selected for the following analyses, including three normal control samples, one precancerous lesion sample, four adenoma samples, and four CRC samples. This dataset was used to identify macrophage-related genes. Additionally, bulk RNA-seq data and corresponding clinical information for CRC patients were retrieved from The Cancer Genome Atlas (TCGA) in January 2024. This dataset included 571 cases of colon adenocarcinoma (COAD) and rectal adenocarcinoma (READ), along with 44 normal control cases. To ensure the accuracy and reliability of the analysis, cases with incomplete clinical information or an OS of less than one month were excluded. Following these criteria, the final TCGA-CRC cohort included 366 COAD and 81 READ samples. Transcripts per million (TPM) values were calculated and subsequently log_2_-transformed to enhance comparability. This cohort was subsequently used to identify genes associated with CRC development.

Moreover, transcriptomic datasets GSE17538 (*n* = 244), GSE17536 (*n* = 177), GSE29621 (*n* = 65), and GSE38832 (*n* = 122) based on the Affymetrix GPL570 platform were also collected from the GEO database. The signal intensities in the three datasets were normalized using the robust multi-array average (RMA) algorithm. Cases with incomplete clinical information or an OS or DSS of less than one month were excluded. The GSE17538 dataset, which includes clinical information (e.g., OS, gender, stage, and age), was used as the training set to construct the prognostic signature. The GSE17536 dataset with clinical information (e.g., OS, gender, stage, and age), the GSE29621 dataset with clinical information (e.g., OS, gender, and stage), along with the GSE38832 dataset with clinical information (e.g., DSS and stage), were used as validation cohorts.

### 4.2. Single-Cell RNA-Seq Analysis

The “Seurat” R package (version 5.0.3) [35] was used for quality control, analysis, and exploration of the scRNA-seq dataset GSE161277. Firstly, according to the study by Zheng et al. [34], cells with fewer than 200 total genes or with a mitochondrial gene proportion greater than 25% were excluded. Cells with fewer than 200 genes are typically considered as low-quality or damaged cells, indicating incomplete transcriptome coverage. Similarly, a mitochondrial gene proportion exceeding 25% suggests that the cell is in a stressed or dying state, likely due to cytoplasmic RNA leakage, leaving predominantly mitochondrial RNA [36]. Genes with an expression level exceeding 200 and expressed in at least three cells were retained. LogNormalize method was applied for data normalizing and the top 2000 highly variable features were identified using the FindVariableFeatures function. Next, principal component analysis (PCA), a linear dimensionality reduction technique, was conducted on the top 2000 highly variable features. The RunHarmony method was employed to correct for batch effects across samples. Based on the elbow plot, 40 principal components were retained. The FindNeighbors and FindClusters functions were then used to cluster these cells. Cell clusters were annotated using the “CellMarker 2.0” (http://bio-bigdata.hrbmu.edu.cn/CellMarker/ (accessed on 26 April 2024)) database, based on the typical biological markers of each cell type.

### 4.3. Construction of a Prognostic Signature Through an Integrative Machine Learning Framework

Given the pivotal role of macrophages in CRC progression, we aimed to develop a MRPS to improve patient outcomes. In detail, macrophage-related genes that were differentially expressed between macrophages and the other cell populations were first identified in the scRNA-seq dataset GSE161277 by using the FindMarkers function with the threshold of |log_2_(FC)| ≥ 0.585 and min.pct = 0.25. Next, in the TCGA-CRC dataset, differential expression analysis was performed between tumor and normal samples using the “DESeq2” R package (version 1.42.1) [37]. The criteria for identifying DEGs were set as |log_2_FC| > 1 and *p*.adj < 0.05, allowing for the identification of genes significantly upregulated or downregulated in CRC samples. To further refine the association of these DEGs with macrophages, the DEGs identified from the bulk RNA-seq dataset were intersected with macrophage-related genes identified from the scRNA-seq dataset. The overlapping genes were referred to as macrophage-related genes in CRC.

To construct a robust prognostic signature with high predictive accuracy, a multi-step approach was employed. Firstly, univariate Cox regression analysis was performed on the TCGA-CRC dataset to screen for macrophage-related genes with potential prognostic significance. Secondly, the GSE17538 dataset was used as the training set, while the TCGA-CRC, GSE17536, GSE38832, and GSE29621 datasets served as validation sets. Ten machine learning methods, including LASSO, Ridge, stepwise Cox, CoxBoost, RSF, Enet, plsRcox, SuperPC, GBM, and survival-SVM, were incorporated, and 101 combinations of these ten algorithms were tested in the GSE17538 training dataset for variable selection and model construction based on a ten-fold cross-validation framework [38]. Thirdly, for each model, the C-index was calculated for both the training and validation sets. The models were then ranked based on their average C-index, with a higher C-index indicating better predictive performance. As a result, the prognostic models with the top highest average C-index value for predicting OS in CRC patients were considered as MPRS candidates for further analysis.

### 4.4. Functional Enrichment Analysis

To explore the potential biological functions of macrophage-related genes in CRC, Gene Ontology (GO) and Kyoto Encyclopedia of Genes and Genomes (KEGG) enrichment analyses were conducted using the “ClusterProfiler” R package (version 4.8.3) [39]. False Discovery Rate (FDR) correction was applied, and an adjusted *p*-value (*p*.adj) < 0.05 was regarded as statistically significant for enrichment.

### 4.5. Predictive Performance Evaluation of the MRPS

To assess the ability of the MRPS for CRC patients, the training and validation sets were stratified into high-risk and low-risk groups based on the median risk score. KM curve analysis was first performed using the “survminer” R package (version 0.5.0) [40] to determine whether there was a significant difference in OS or DSS between the two risk groups. The log-rank test was applied to assess statistical significance, with a *p*-value threshold set at less than 0.05. Subsequently, ROC curve analysis was conducted using the “timeROC” R package (version 0.4) [41] to evaluate the sensitivity and specificity of the prognostic model in predicting OS or DSS for CRC patients.

### 4.6. Independence Assessment of the MRPS Ability

To evaluate the independent prognostic value of the MRPS, univariate and multivariate Cox regression analyses were conducted in the GSE17538, GSE17536, GSE29621, GSE38832, and TCGA-CRC datasets. Univariate Cox regression analysis was first performed to assess the association between risk factors (such as risk score, stage, gender, and age) and prognosis of CRC patients. This was followed by multivariate Cox regression analysis to examine whether the MRPS-derived risk score and clinical characteristics were independent predictors of OS or DSS. HRs and 95% confidence intervals (CIs) were calculated for each variable, with *p* < 0.05 considered statistically significant.

### 4.7. Performance Comparison of the MRPS with Established Models in CRC

To further evaluate the performance of the MRPS, we comprehensively retrieved published prognostic signatures for CRC from the PubMed database (Table S2). The C-index was employed as the primary metric for comparison. Firstly, for each model, the risk scores were calculated for each sample across all cohorts by utilizing gene expression levels and their respective coefficients. Secondly, in the datasets such as GSE17538, GSE17536, GSE29621, and TCGA-CRC, the OS time and survival status data of patients were taken as the response variables, with the risk scores used as the predictor variables to construct the Cox proportional hazards model. In the GSE38832 datasets, DSS was used as the response variable instead of OS due to the unavailability of OS data for this cohort. The coxph function was employed to calculate the C-index for evaluating model performance. Finally, the statistical significance of differences in the C-index values between published models and the MRPS was assessed using the “compareC” R package (version 1.3.2).

### 4.8. Construction and Validation of an MRPS-Based Nomogram for CRC

The “rms” R package (version 6.8.1) [42] was used to construct a nomogram by incorporating the prognostic signature-derived risk score and common clinical characteristics (i.e., age and stage) in the GSE17538 dataset. Then, time-dependent ROC curve analysis was performed using the “timeROC” R package (version 0.4) [41] to evaluate the discriminative ability of the nomogram-derived score in predicting the 1-, 2-, and 3-year OS of CRC patients. The AUC value served as a quantitative measure, with values closer to 1 indicating stronger discriminative ability. Furthermore, calibration curves were utilized to assess the accuracy of the model. These curves graphically displayed the predictive accuracy of the model by comparing the predicted probabilities with the actual observed outcomes. In addition, DCA was performed to evaluate the performance of the nomogram by calculating its net clinical benefit.

### 4.9. Comprehensive Analysis of Immune Cell Infiltration in High- and Low-Risk Groups

To further analyze the differences in immune cell infiltration between high-risk and low-risk groups, this study employed two methods: ssGSEA and CIBERSORT. Firstly, using the “GSVA” R package (version 1.48.3) [43], ssGSEA was performed on 28 immune cell-related gene sets based on gene expression data from the GSE17538 dataset. Enrichment scores for each gene set were calculated to infer the relative activity of immune cells in the samples. Then, the CIBERSORT algorithm, utilizing a support vector regression model and immune cell signature profiles (such as LM22), was applied to estimate the relative abundance of 22 immune cell subtypes from the GSE17538 dataset. To uncover potential associations between gene expression levels and immune cell infiltration, Spearman correlation analysis was performed. The *p*-value < 0.05 was set as the threshold for statistical significance. Finally, visualizations were generated using the “ggplot2” R package (version 3.5.1) to display correlation heatmaps and box plots of immune cell infiltration differences, offering a clear representation of the variations in the immune microenvironment between the high-risk and low-risk groups.

### 4.10. Correlation Analysis of the Prognostic Model and Response to Immune Checkpoint Inhibitor Therapy

To investigate the expression levels of immune checkpoint genes between high-risk and low-risk groups, a set of 47 immune checkpoint genes related to CRC was collected (Table S3) [44]. The mRNA expression data for these genes were then extracted from the GSE17538 dataset. Statistical comparisons of gene expression between high-risk and low-risk groups were performed using the Wilcoxon rank-sum test. Additionally, Pearson correlation analysis was conducted to assess the relationship between the expression levels of these immune checkpoint genes and the patients’ risk scores. The *p*-value < 0.05 was used to determine statistical significance.

### 4.11. Screening Potential Drugs for CRC Treatment Based on the GDSC, CTRP, and PRISM Databases

To evaluate the chemotherapy response between high-risk and low-risk CRC patient groups, drug sensitivity prediction analyses were conducted. The “oncoPredict” R package (version 1.2) [45] was employed to match the gene expression profiles of CRC patients in the GSE17538 dataset to the expression profiles of cancer cell lines in the GDSC database (https://www.cancerrxgene.org/ (accessed on 13 August 2024)), to calculate the half-maximal inhibitory concentration (IC_50_). In addition, the “pRRophetic” R package (version 0.5) [46] was utilized to fit gene expression profiles of CRC patients in the GSE17538 dataset to the drug sensitivity data of human cancer cell lines from the CTRP(http://portals.broadinstitute.org/ctrp/ (accessed on 13 August 2024)) and PRISM (https://depmap.org/portal/prism/ (accessed on 13 August 2024)) databases to calculate the AUC values [47]. Statistical differences in drug sensitivity between high-risk and low-risk groups were evaluated using Wilcoxon tests, with a *p*-value < 0.05 considered significant. Pearson correlation analysis was further conducted to identify drugs negatively correlated with risk scores, which were then regarded as potential therapeutic compounds for CRC patients.

To comprehensively evaluate the safety and efficacy of candidate drugs and understand their mechanisms of action in the human body, the ADMET properties of the candidate drugs were analyzed using the online ADMETlab 2.0 (https://admetmesh.scbdd.com/ (accessed on 28 August 2024)) and SwissADME (http://www.swissadme.ch/ (accessed on 28 August 2024)) databases.

### 4.12. Analysis of Tumor Mutation Burden Across Different Risk Groups

To explore the differences in TMB values between high-risk and low-risk groups, somatic mutation data for CRC patients were obtained from the TCGA database. The somatic mutation characteristics between different risk groups were then analyzed by the “maftools” R package (version 2.18.0) [48]. Furthermore, the TMB for each patient was calculated and then all CRC patients were divided into low or high TMB groups according to TMB median value. A violin plot illustrating the TMB distribution between the high-risk and low-risk groups was generated using the ggviolin function. Statistical differences in TMB values between the two groups were assessed, with a *p*-value < 0.05 indicating significance. To evaluate the effectiveness of combining the signature-derived risk score with the TMB in distinguishing the survival probabilities, CRC patients were divided into four subgroups: high TMB + high risk, high TMB + low risk, low TMB + high risk, and low TMB + low risk. KM survival analysis was then performed using the “survival” R package (version 3.7.0) to assess the differences in OS among the four subgroups.

### 4.13. Pseudo-Temporal Analysis of Macrophage Subpopulations in CRC

To explore the classification of macrophage subpopulations and their functional differences, sub-clustering analysis of identified macrophages was performed using the “Seurat” R package (version 5.0.3) [35]. Subsequently, pseudotime trajectory analysis was conducted using the “monocle” R package (version 2.28.0) to investigate the differentiation trajectories of macrophage subpopulations [49]. The expression dynamics of MRPS-related genes at different pseudotime stages were further analyzed to uncover their potential roles in macrophage development [50].

### 4.14. Statistical Analysis

All statistical analyses were conducted using the R software (versions: R 4.3.2). The Benjamini–Hochberg (BH) method was applied to estimate the FDR for multiple testing adjustments. DEGs between tumor and normal control samples were identified with *p*.adj < 0.05. Enriched GO or KEGG items were identified by using the threshold of *p*.adj < 0.05. Kaplan–Meier survival analysis and log-rank tests were performed using the “survival” package in R (version 3.5.7) to compare OS or DSS between high- and low-risk groups. Univariate and multivariate Cox regression analyses were employed to investigate independent prognostic factors. Spearman’s correlation analysis was utilized to explore the relationship between risk scores and immune cell infiltration.

## 5. Conclusions

In summary, by integrating scRNA-seq and bulk RNA-seq technologies, we constructed and validated an MRPS, which serves as a promising tool for predicting and personalizing treatment for CRC patients. Drug sensitivity analysis identified five promising agents for high-risk patients, including Mitoxantrone, Bortezomib, Teniposide, YM-155, and PLX-4720. Our findings highlight the prognostic value of the MRPS and the critical role of macrophages in tumor progression, immune modulation, and treatment response. Future studies with larger sample sizes and functional validation are needed to further enhance the clinical utility of the MRPS.

## Data Availability

The dataset provided in this study can be downloaded from the online website TCGA-CRC (https://portal.gdc.cancer.gov/ (accessed on 18 January 2024) GEO: https://www.ncbi.nlm.nih.gov/geo/ (accessed on 3 April 2024). **Use of Artificial Intelligence:** We did use ChatGPT-4.0 to assist in enhancing the language and clarity of the manuscript. Specifically, ChatGPT-4.0 was employed to refine the writing style, improve sentence structure, and ensure that the manuscript met the language standards suitable for publication. The content and scientific analysis, however, were entirely prepared and reviewed by the authors.

## Supplementary Materials

The following supporting information can be downloaded at: https://www.mdpi.com/article/10.3390/ijms26020811/s1.
