# Supplementary material for: Integrative Single-Cell and Bulk RNA Sequencing Identifies a Macrophage-Related Prognostic Signature for Predicting Prognosis and Therapy Responses in Colorectal Cancer
Source: Int J Mol Sci. 2025 Jan 19;26(2):811. doi: 10.3390/ijms26020811 (PMC11765994; doi:10.3390/ijms26020811)
Supplement: Supplementary file 1 [file ijms-26-00811-s001.zip › Supplementary figures.pdf]

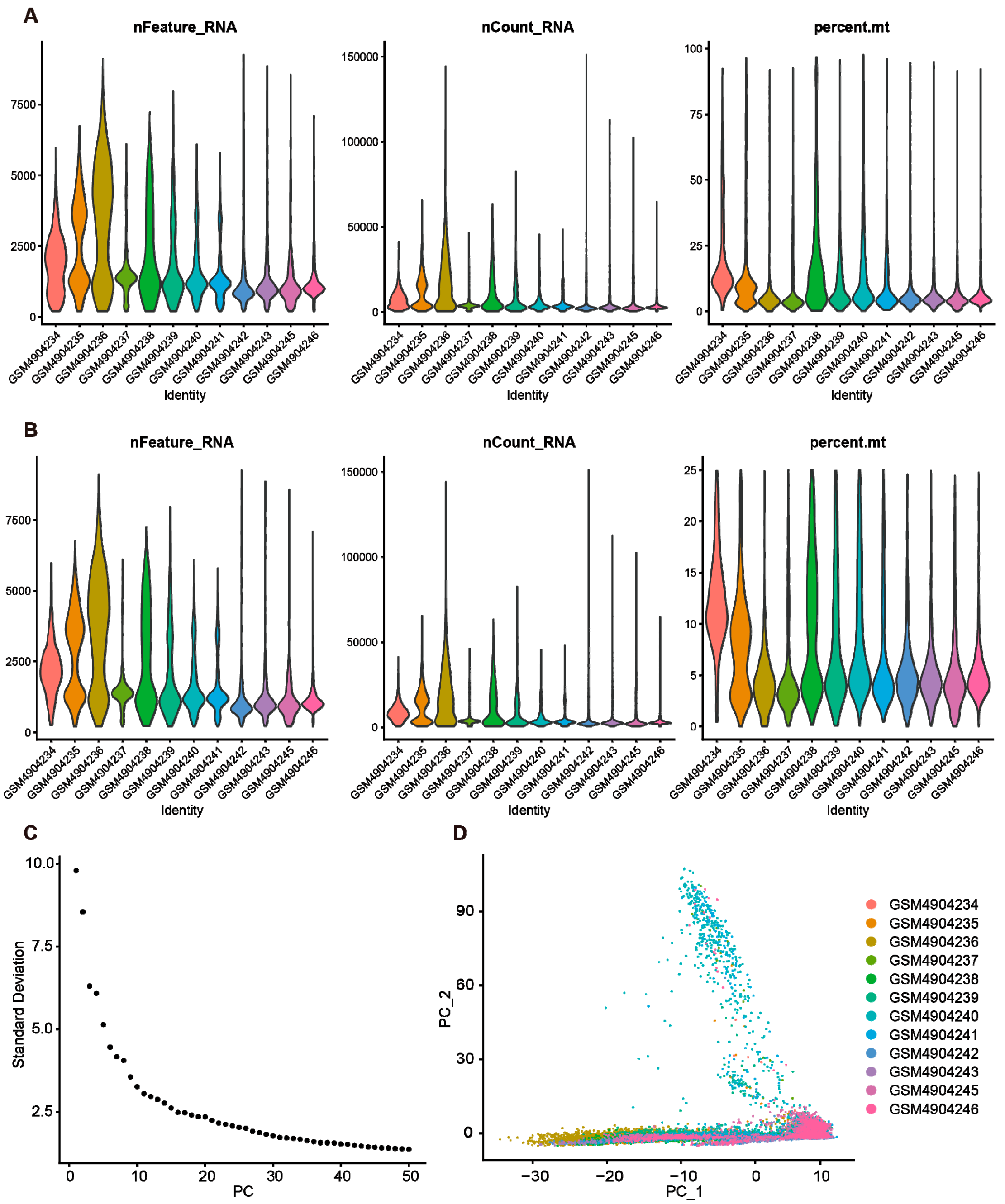

Figure S1. scRNA-seq analysis of CRC samples. (A, B) The distribution of the total number of expressed genes (nFeature_RNA), the number of transcripts (nCount_RNA), and the proportion of mitochondrial transcript expression (percent.mt) in single-cell data from 12 CRC samples before quality control (A) and after quality control (B). (C) Elbow plot showing the standard deviation of each principal component (PC). (D) PCA plot of the 12 samples.

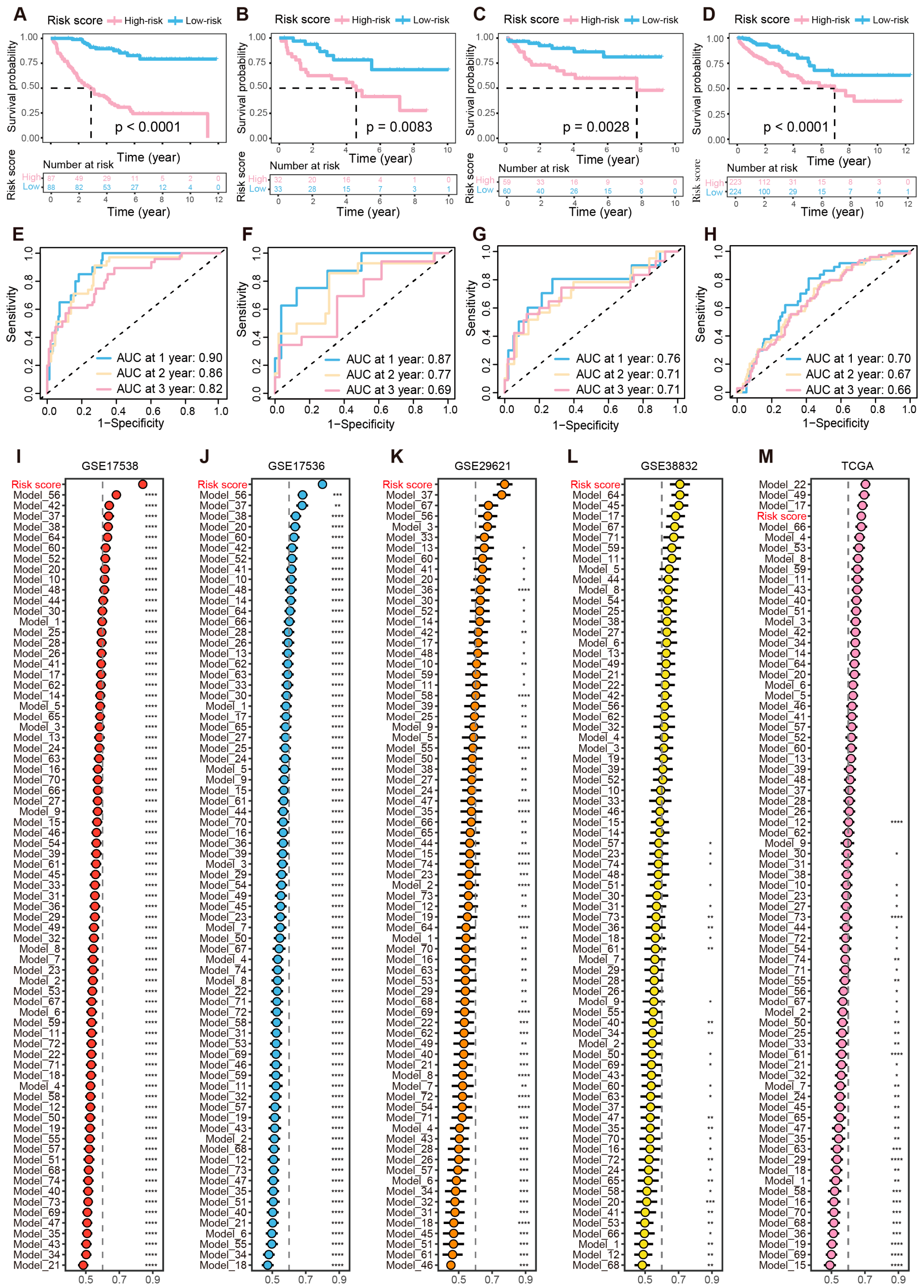

Figure S2. Performance evaluation of MRPS in CRC. (A-D) KM survival curves for OS or DSS of CRC patients in the high-risk and low-risk groups across GSE17536 (A), GSE29621 (B), GSE38832 (C), and TCGA (D) datasets. (E-H) ROC curves for OS or DSS at 1-year, 2-year, and 3-year in GSE17536 (E), GSE29621 (F), GSE38832 (G), and TCGA datasets (H). (I-M)

Performance comparison between MRPS and other reported models in the GSE17538 (I), TCGA (J), GSE17536 (K), GSE29621 (L), and GSE38832 datasets (M). $^{*}P<0.05$; $^{**}P<0.01$; $^{***}P<0.001$; $^{****}P<0.0001$.

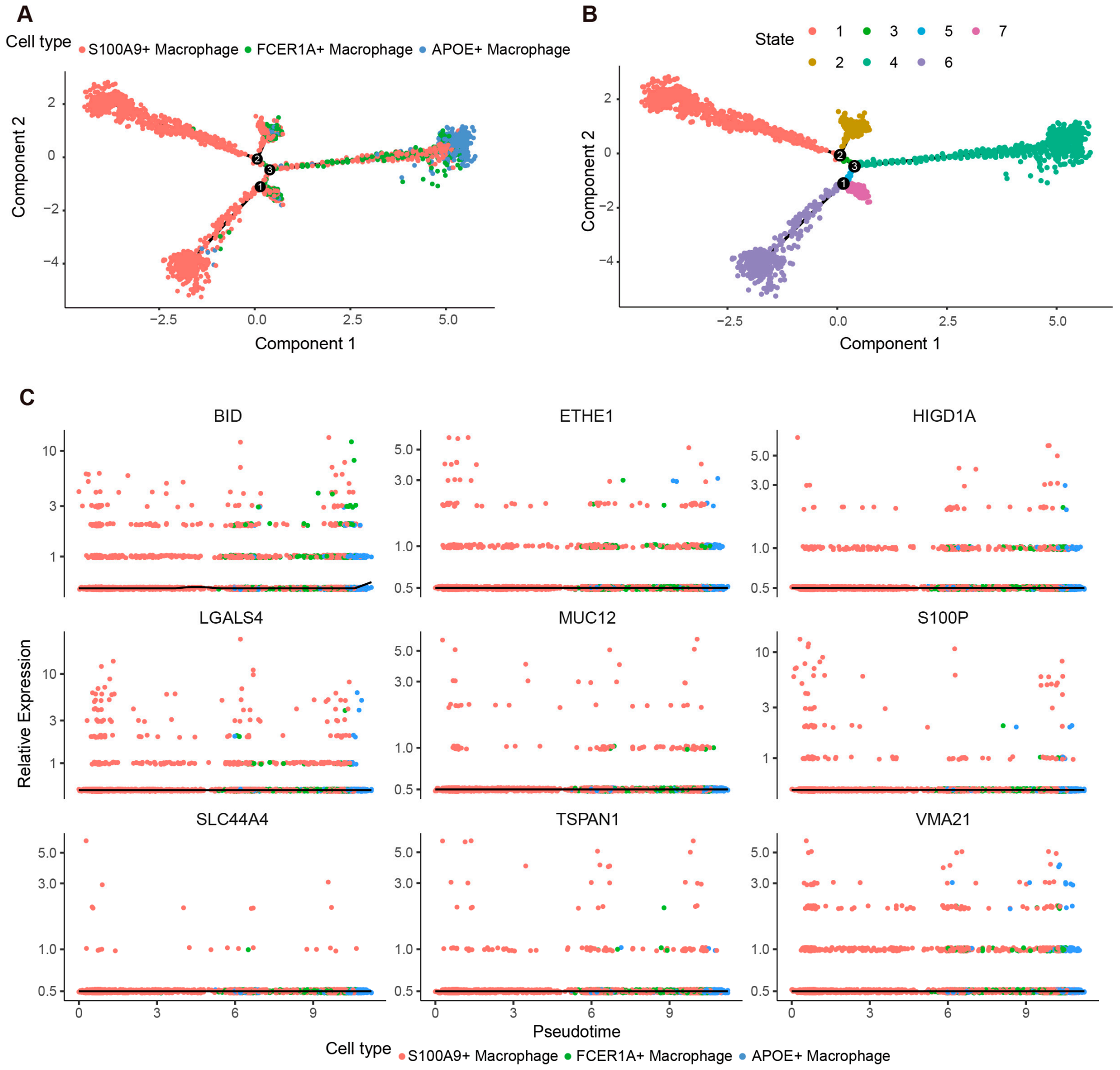

Figure S3. Pseudotime trajectory analysis of macrophage subclusters. (A) Distribution of the APOE^{+}, FCER1A^{+}, and S100A9^{+} macrophage subclusters along the developmental trajectory. (B) The pseudotime trajectory coloring by state of macrophage subclusters. (C) The expression levels of BID, ETHE1, HIGD1A, LGALS4, MUC12, S100P, SLC44A4, TSPAN1, and VMA21 along the pseudotime trajectory across the APOE^{+}, FCER1A^{+}, and S100A9^{+} macrophage subclusters.
